# Epidemiology of Injuries in Ultimate (Frisbee): A Systematic Review

**DOI:** 10.3390/sports8120168

**Published:** 2020-12-21

**Authors:** Diana Fajardo Pulido, Reidar P. Lystad

**Affiliations:** Australian Institute of Health Innovation, Macquarie University, Sydney 2109, Australia; reidar.lystad@mq.edu.au

**Keywords:** sports injury, flying disc, incidence, prevalence, risk factors, concussion

## Abstract

Ultimate is a high-intensity, non-contact team sport played with a flying disc (e.g., frisbee). Despite the growing popularity of ultimate worldwide, there is limited information about the epidemiology of injury in the sport. The purpose of this review is to provide a comprehensive overview and synthesis of the literature on the epidemiology of injury in ultimate. A comprehensive search of the literature was conducted in five electronic databases (i.e., MEDLINE, Embase, AMED, SPORTDiscus, and AusportMed). All databases were searched from inception to 1 July 2020. A total of eleven studies were included and qualitatively synthesized. Injury incidence rate estimates ranged from 0.4 to 84.9 injuries per 1000 athlete-exposures. The lifetime prevalence of any injury and concussion were 100% and 26%, respectively. The most commonly injured body region was the lower limb, with the knee and thigh being the most frequently injured anatomical locations. The most frequent injury types were muscle injuries and superficial contusions. The most common injury situation was direct contact with another player. There is a substantial risk of injury in ultimate, in particular muscle strains and joint sprains to the knee and shoulder areas. Development and implementation of effective, sport-specific injury prevention initiatives, including improved injury risk management and sport safety culture, should be a priority to reduce the burden of injury in ultimate.

## 1. Introduction

Flying disc sports is an umbrella term for a diverse range of sports played with a flying disc (e.g., frisbee), including ultimate, guts, disc golf, and discathon [1]. The most popular among these is ultimate, which is played on a field of 100 m by 37 m (i.e., approximately the same length but half the width of a soccer pitch), including 18 m end-zones at each end [2]. Two teams, each of which comprising a maximum of seven on-field players, aim to score a goal in the opposing team’s end-zone [2]. During the game, the disc is advanced through the field by the offensive team throwing it between players while the defensive team is trying to intercept and take possession of the disc [2]. Players are not allowed to run while holding the disc; when obtaining possession of the disc, players must establish a pivot foot and release the disc within 10 seconds [2]. If the disc is knocked down, intercepted, or lands outside the field, the opposing team takes possession of it and the direction of play reverses [3]. A goal is scored when a player on the offensive team receives the disc in the opposing team’s end-zone. There is a five-minute break at half-time, which occurs when one team has scored eight goals [2], and the teams switch end-zones before resuming play. Teams are allowed unlimited substitutions while the game is paused (i.e., after a goal is scored and during half-time) [2]. Games last until a team has scored 15 goals, typically around 100 min [2]. Ultimate has three competition divisions: Women’s, men’s, and mixed with different levels of play (e.g., local, regional, national, and international). An interesting feature of ultimate is that the sport is self-refereed with a great emphasis on fair play referred to as “spirit of the game”. During the game, players themselves act as referees mediating any call of foul or violation, which is settled according to the rules of the game [2].

Participation in local, national, and international ultimate competitions is rapidly increasing worldwide. The World Flying Disc Federation (WFDF) reported an annual growth rate of 11% during the last decade, with more than 170,000 active members worldwide [4]. The governing body for the sport in the United States, USA Ultimate, reported more than 850,000 members in 2019 [5]. As a testament to its growing popularity, ultimate was recognized as a sport by the International Olympic Committee (IOC) in 2015 and is potentially eligible for inclusion in future Olympic Games.

Ultimate is a high-intensity, non-contact sport with high physical demands [6], and, as in any sport, there is an inherent risk of injury [7,8]. Because injury can result in reduced performance, absence from sports participation, limitation of activities of daily living, and permanent disability, prevention of sports injuries should be a priority [9]. The theoretical framework for sports injury prevention suggests that research progresses in a stepwise manner from problem identification to adoption of effective interventions [10]. Specifically, this framework includes the following steps: (1) Establishing the extent of the injury problem; (2) establishing the causes and mechanisms of injury, including identification of risk factors; (3) developing and introducing preventative measures; and (4) assessing the efficacy/effectiveness by repeating the first step.

In regard to establishing the causes and mechanisms of sports injury, it is important to account for all the factors involved [11,12], including: intrinsic risk factors (e.g., age and previous injury history); extrinsic risk factors (e.g., game conditions, playing surface, and level of importance of a game); and factors related to the inciting event such as playing situation (i.e., throwing skills, cutting, and fatigue), athlete and opponent behavior (i.e., jumping, collisions, and diving), and body biomechanics (i.e., sprinting and pivoting).

There has been no comprehensive synthesis of the literature on injuries in ultimate to date. The purpose of our systematic review, therefore, was to provide a comprehensive overview and synthesis of the epidemiology of injuries in ultimate. Specifically, the objectives of our review were: (1) To determine the incidence and prevalence of injury; (2) to describe the injury pattern in terms of the distribution of injury by body region, type of injury, mechanism of injury, mode of onset, and injury severity; and (3) to identify risk factors for injury.

## 2. Materials and Methods

This systematic review adhered to the guidelines in the Preferred Reported Items for Systematic Reviews and Meta-analysis (PRISMA) Statement [13].

### 2.1. Protocol and Registration

The protocol for this review was registered in the international prospective register of systematic reviews PROSPERO (registration number CRD42018110863).

### 2.2. Eligibility Criteria

This review included peer-reviewed journal articles reporting on prospective and retrospective cohort, cross-sectional, and case-control studies. Articles from non-peer reviewed sources and case studies, case reports, case series, review articles, commentaries, editorials, opinion pieces, and letters to the editor were excluded from this review. Eligible studies had to investigate injuries sustained during ultimate training or competition, and report on incidence or prevalence, injury pattern (e.g., distribution of injury by body region, type of injury, mechanism of injury, mode of onset, and injury severity), or risk factors. There were no restrictions based on the language of publication, date of publication, geographical location of study, age of participants, gender of participants, or level of play. This review did not exclude any studies on the basis of operational definition of a reportable injury.

### 2.3. Search Strategy

A comprehensive search of the literature was conducted using electronic searching of MEDLINE, Embase, and AMED databases via the Ovid platform, SPORTDiscus database via the EBSCOhost portal, and AusportMed database via the Informit platform. All databases were searched from inception to 1 July 2020. The key concepts used in the electronic searches were “injury”, including synonyms (e.g., wound or trauma), and “ultimate”, including synonyms (e.g., frisbee or flying disc). See Supplementary Material Table S1 for the full search string for the MEDLINE database. In addition, we hand-searched reference lists of included studies and relevant review articles to identify potentially eligible articles not captured by the electronic searching.

### 2.4. Study Selection

Records identified through electronic database searching were combined and de-duplicated in EndNote X9.1 (Clarivate Analytics, Philadelphia, United States). Two independent reviewers screened titles and abstracts to discard irrelevant records that clearly did not meet the eligibility criteria. Full-text articles of the remaining potentially eligible studies were retrieved and subsequently assessed against the eligibility criteria by two independent reviewers. Any disagreement between reviewers was resolved by mutual consensus.

### 2.5. Assessment of Study Quality

Included studies were evaluated by two independent reviewers using the Strengthening the Reporting of OBservational studies in Epidemiology (STROBE) Statement [14]. It is important to note that the STROBE Statement checklist was designed to help improving the quality of reporting rather than directly evaluating study quality. However, in the absence of a single, gold-standard tool for evaluating the quality of epidemiological studies, the STROBE Statement may suffice as a starting point [15]. Authors have previously used the STROBE Statement checklist to evaluate study quality [16,17], whereby studies were categorized as either poor, moderate, or good quality based on the percentage of fulfilled items (e.g., <50%, 50% to 80%, and ≥80%, respectively). Discrepancies between reviewers were resolved by mutual consensus.

### 2.6. Data Extraction and Synthesis

The following data were extracted and tabulated in an electronic spreadsheet: (1) Study characteristics (i.e., author names, publication year, study design, country, setting, and study period); (2) study population (i.e., sample size and demographics); (3) injury epidemiology data (i.e., incidence or prevalence; distribution by body region, type, mechanism, mode of onset, and severity; and risk factor data). 

Extracted data were qualitatively synthesized and summarized. One athlete-exposure was defined as one athlete being exposed to the possibility of incurring an injury while participating in a single ultimate game or training session. If not explicitly reported, injury incidence rates per 1000 athlete-exposures and injury incidence rate ratios per 1000 athlete-exposures were calculated from the available data whenever possible. Injury incidence rates and rate ratios were calculated with 95% confidence intervals using standard formulae for Poisson rates [18]. Injuries were categorized by body region, type, mechanism, mode of onset, and severity in accordance with classifications and recommendations in the recent IOC consensus statement on methods for recording and reporting of epidemiological data on injury and illness in sport [19].

## 3. Results

### 3.1. Study Selection

**Figure 1:**
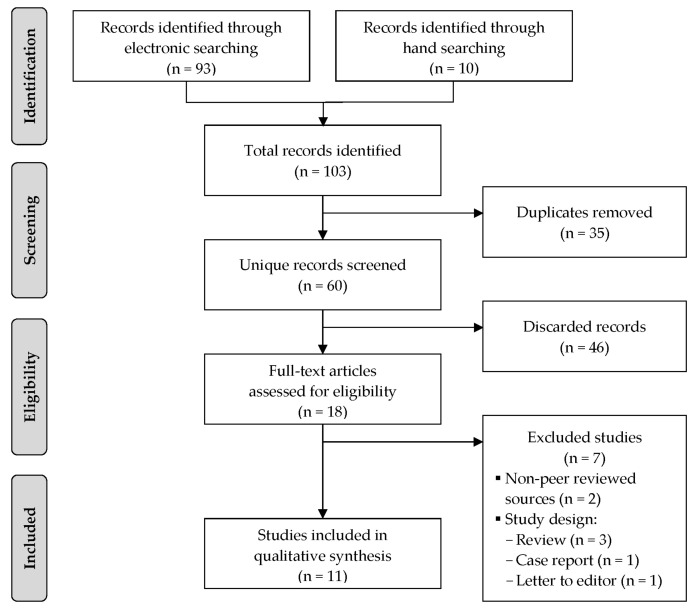
PRISMA flowchart of the study selection process.

A total of 103 records were identified through electronic database and hand-searching. After screening the titles and abstracts, full-text versions of 16 potentially eligible studies were evaluated against the eligibility criteria. Seven studies [7,8,20,21,22,23,24] were excluded due to the following reasons: Non-peer-reviewed articles (i.e., magazine article, n = 1; conference abstract, n = 1) and ineligible study design (i.e., review article, n = 3; case report, n = 1; letter to the editor, n = 1). Thus, a total of 11 studies [3,25,26,27,28,29,30,31,32,33,34] were included in this systematic review. Figure 1 shows a PRISMA flow chart of the study selection process. The corresponding authors of five included studies [25,30,31,34] were contacted via email to seek further clarification of their reported data, with the requested clarifications being obtained for two of the studies [25,30].

### 3.2. Characteristics of Included Studies

The 11 included studies comprised 7 prospective cohort studies [3,25,28,30,31,32,33], 1 retrospective cohort study [34], and 3 cross-sectional studies [26,27,29]. Nine of the included studies were conducted in the United States [25,27,28,29,30,31,32,33,34], one was conducted in Poland [26], and one was conducted in five different countries [3]. The data were obtained from World Championships [3], collegiate or university championships, leagues, or series [25,28,30,31,33,34], regional tournaments [29], local club sports [26], national leagues, and tournaments [27,32]. The characteristics of the included studies are summarized in Table 1 and a Graphical Overview for Evidence Reviews (GOfER) diagram is provided in Supplementary Material Figure S1. The operational injury definition varied across the included studies, including incident resulting in medical attention or care or absence from participation in training or game play. One study [27] investigated concussion injuries only. It is important to highlight that when an injury occurs during an ultimate game, depending on the severity of the injury, the players involved can call an injury stoppage where the game is stopped for a maximum of 75 seconds before play resumes, or an injury time-out where the game is suspended while the injured player is substituted [2]. The methodological quality of the included studies ranged from poor [28] to moderate [3,25,34] to good [26,27,29,30,31,32,33]. A detailed overview of the quality assessment of each included study is provided in Supplementary Material Table S2.

### 3.3. Injury Incidence and Prevalence

The reporting of injury incidence and prevalence varied considerably among the included studies. Of the eight cohort studies, three studies did not report on injury incidence [3,25,28], while three studies [30,31,32] reported injury time-out incidence rates per 1000 athlete-exposures ranging from 12.6 to 33.4 to 84.9 and two studies reported [33,34] found time-loss injury incidence rates per 1000 athlete-exposures ranging from 0.4 to 10.1. Three of these studies [30,32,34] reported separate incidence rates for training (10.1 and 20.0 injury time-outs per 1000 athlete-exposures; 0.4 time-loss injuries per 1000 athlete-exposures) and competition (14.5 and 45.1 injury time-outs per 1000 athlete-exposures; 1.3 time-loss injuries per 1000 athlete-exposures). Of the three cross-sectional studies, two studies [26,29] found the lifetime prevalence of injury to be 100%, while one study [27] reported the lifetime prevalence of concussion to be 26%.

### 3.4. Injury Pattern

Six studies reported on distribution of injuries by body region and anatomical location [3,25,26,30,31,32] (Table 2). The most commonly injured body region was the lower limb (range: 27.0% to 88.1%), followed by the upper limb (range: 4.2% to 18.1%) and the head and neck (range: 3.5% to 15.4%). Among lower limb injuries, the most frequently injured anatomical areas were the knee (range: 19.5% to 39.7%), thigh (range: 11.9% to 31.9%), and ankle (range: 15.5% to 30.1%). Among upper limb injuries, the most commonly injured anatomical area were the wrist and hand (range: 30.1% to 52.9%), shoulder (range: 17.6% to 50.0%), and elbow (range: 9.1% to 29.4%).

Four studies reported on the distribution of injuries by type of injury [3,26,30,32] (Table 3). The most common type of injury was muscle injury (range: 24.2% to 36.9%), followed by joint sprain (range: 7.6% to 49.1%), and superficial contusion (e.g., bruising and hematoma) (range: 10.6% to 22.7%).

Four studies reported on injury mechanism [27,30,31,32] (Table 4). The most common mechanism of injury was non-contact (range: 40.4% to 68.6%), followed by direct contact with another athlete (range: 30.5% to 39.4%) and direct contact with an object (range: 0.7% to 20.2%). One study [28] found that the vast majority (97.0%) of concussions were caused by either indirect contact through another athlete or indirect contact through an object (50.9% and 46.1%, respectively).

Two studies reported mode onset of injury [30,32] (Table 5). In both studies, the vast majority of injuries were acute, sudden onset injuries (79.7% and 92.0%), while the remaining injuries were repetitive, gradual onset injuries (20.3% and 8.0%).

None of the included studies reported any data on injury severity.

### 3.5. Risk Factors for Injury

Four studies reported on risk factors for injury [27,30,32,33]. There was conflicting evidence regarding gender as a risk factor. For instance, Brezinski and colleagues [33] found no significant difference in time-loss injury incidence rates between males and females (RR 1.15, 95% CI 0.44–2.95). However, Swedler and colleagues [30] reported that, compared to female athletes, males were more likely to sustain shoulder injuries (RR 1.61, 95% CI 1.01–2.60), wrist injuries (RR 3.27, 95% CI 1.18–11.83), and injuries on a layout (RR 1.69, 95% CI 1.23–2.34). Lazar and colleagues [29] found no differences between males and females in lifetime prevalence of concussion (Χ^2^ = 0.28, P = 0.59). Both Swedler et al. [30] and Hess et al. [32] found that athletes were more likely to be injured during competition than training (RR 1.43, 95% CI 1.28–1.61 and RR 2.25, 95% CI 1.95–2.58, respectively). Hess and colleagues [32] found that athletes sustained more injuries when playing on artificial turf compared to natural grass (RR 1.34, 95% CI 1.17–1.53). They also reported a higher injury incidence rate when playing on a wet surface than a dry surface, but the difference was not statistically significant (RR 1.47, 95% CI 0.98–2.11). Neither did they find any differences for athletes playing on back-to-back days or doubleheader versus athletes playing a single game in a week (RR 0.94, 95% CI 0.70–1.27) [32].

## 4. Discussion

This is the first systematic review of the epidemiology of injuries in ultimate athletes. It highlights a substantial injury problem in the sport, with muscle strains and joint sprains to the knee, thigh, ankle, and shoulder areas being predominant and concussion being unexpectedly common. This review also identifies major gaps in the literature and provides recommendations for future research.

### 4.1. Injury Incidence and Prevalence

There are limited good quality data estimating the extent of the injury problem in ultimate. Although a few studies revealed a lifetime (or long-term period) prevalence of injury of 100% among ultimate athletes [25,26,29], this information is not particularly informative and of very limited utility when it comes to assessing the risk of injury in ultimate and compare it with other sports. Among the three prospective cohort studies that reported exposure-adjusted injury incidence rates [30,31,32,33], the estimates varied considerably, from 10 to 85 injuries per 1000 athlete-exposures. The reason for this variability is unclear. Although all studies used a similar operational injury definition (i.e., injury time-out or time-loss injury), there were differences in exposure definitions. Yen and colleagues [31] observed games during a single, three-day tournament, Brezinski and colleagues [33] collected competition data from a single university club during a 16-week semester, while Swedler and colleagues [30] and Hess and colleagues [32] collected data from competition and training during an entire season in a national professional league and college series, respectively. However, the inclusion of training injury data in the latter two studies appear to account for only a small amount of the observed variability in overall injury incidence rates, which suggests that the observed variability is more likely due to other methodological differences.

The injury incidence rate in ultimate is similar to other team sports involving high-intensity running. For instance, the injury incidence rate during competition in lacrosse has been reported at 13 per 1000 athlete-exposures for male youth and adult athletes [34,35] and 7 per 1000 athlete-exposures for female adult athletes [35,36], while the injury incidence rate in field hockey has been reported to be 7 per 1000 athlete-exposures in female youth and adult athletes [36,37]. Despite being a non-contact sport with an emphasis on fair play, the substantial injury problem in ultimate highlighted in this review suggests there is an urgent need to develop and implement injury prevention initiatives in the sport, including better enforcement of the rules of the game to avoid contact and collision among athletes.

This review found revealed an unexpected and concerning concussion problem in ultimate, with reported incidence rates ranging from 0.42 to 0.45 per 1000 athlete-exposures [30,32] and lifetime prevalence of 26% [27]. These incidence and prevalence estimates are higher than those reported in other non- and limited-contact sports [38,39,40]. For example, concussion incidence rates in volleyball and softball have been reported to be 0.18 and 0.19 per 1000 athlete-exposures, respectively [40]; and the lifetime prevalence of concussion in non- and limited-contact sports has been reported to be 18% and 20%, respectively [41]. Perhaps even more concerning is that contrary to the international consensus statement on concussion in sport [42], 43% of concussed ultimate athletes reported returning to play in the same game they sustained their concussion [27]. The majority of concussions in ultimate result from direct contact with another player or direct contact with an object (i.e., ground), which is consistent with reports from studies in other non- and limited-contact sports [43,44]. This suggest that there is an urgent need for developing and implementing injury prevention strategies to reduce the occurrence and mitigate the consequences of concussion in ultimate. In particular, sport governing bodies for ultimate are strongly encouraged to adopt evidence-based guidelines for concussion recognition, evaluation, management, and return to play.

### 4.2. Injury Pattern

The injury pattern in ultimate is, unsurprisingly, similar to that in other team sports involving high-intensity running and an active use of upper limbs (e.g., basketball, lacrosse, field hockey, and handball) and other flying disc sports (e.g., flying disc golf). That is, muscle strains and joint sprains to the knee, thigh, ankle, and shoulder areas were predominant. Previous studies investigating the injury pattern in team sports involving high-intensity running sports have found that around 90% of injuries involved lower extremities [45,46], and of these, muscle injuries and ankle sprains are the most common type of injuries [45,46]. Among team sports that combine high-intensity running and active use of the upper limb, shoulder injuries are common [36,47,48,49], with at least 30% of handball players reporting current shoulder pain [50]. Similarly, in lacrosse, around 20% of participants reported injury of the upper limb, with the most commonly injured anatomical locations being the wrist and hand and shoulder [51]. In flying disc golf, 43% of players reported previous shoulder injury, while 28% and 21% reported previous knee and ankle injuries, respectively [52]. Because knee injury is a major contributor to temporary incapacity and long-term disability [53], developing and implementing injury prevention interventions focusing on the lower limb in general, and the knee in particular, should be a priority in ultimate. Existing injury prevention programs such as FIFA 11+ has demonstrated to be effective in reduce risk of injury [54], and could be easily adapted for use among ultimate athletes.

**Figure 2:**
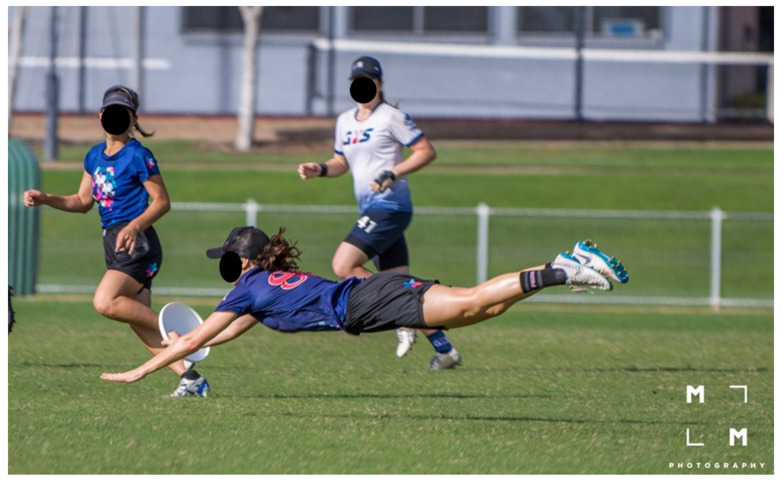
Athlete demonstrating a properly executed layout. Reproduced with permission from Mark Milne, MM Photography.

**Figure 3:**
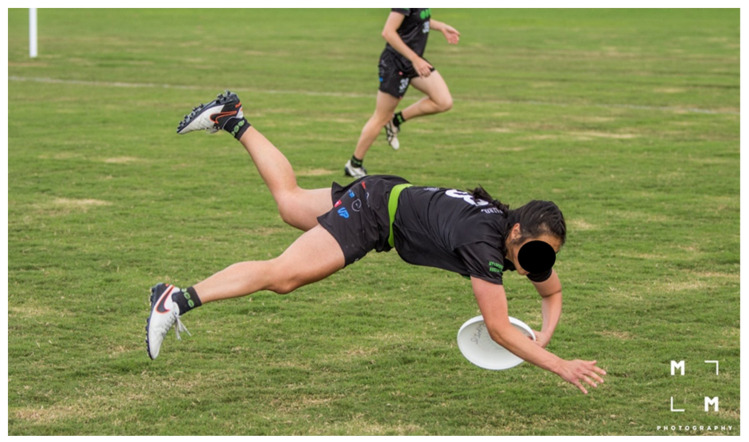
Athlete demonstrating a poorly executed layout. Reproduced with permission from Mark Milne, MM Photography.

The most common mechanism of injury was non-contact (e.g., running, laying out, and jumping). In ultimate, intermittent running performance and sudden jumping can influence the cardiovascular loading of athletes, which may increase fatigue and the risk of injury without collision [6], this pattern has also been reported in other competitive team sports where it has been seen an increased risk of injury while running in defensive or offensive positions and jumping [9,53,55]. The second most common injury mechanism was direct contact with another athlete, with layout and jumping being a common situation for injury. Layout is a unique feature of ultimate that requires training and technique [3]. While aiming to reach the disc, at the end of the run, the player dives while maintaining legs and arms fully extended and the body parallel to the ground, and as the disc is caught, the body lands on the ground [3] (Figure 2). If a layout is poorly executed, the contact with the ground can cause injuries to the head, shoulder, or elbow [3] (Figure 3). Layout is used in both defensive or offensive positions and exposed players to a high risk of collision with an opposing player. A video clip showing layouts executed from both defensive and offensive positions is provided in Supplementary Material Video S1 (reproduced with permission from Gerom Amurao). Further research is needed to investigate layout-related injury mechanisms in more detail and to identify develop targeted prevention strategies.

### 4.3. Risk Factors

This review highlights a paucity of data identifying risk factors for injury in ultimate. Currently, there is insufficient and inconsistent evidence to suggest that the injury risk is different for females and males. There is, however, evidence from two studies indicating that the risk of injury is significantly greater in competition than in training. This finding is unsurprising and consistent with reports from previous studies in other team sports such as handball [49], soccer [17], and basketball [56].

### 4.4. Strengths and Limitations

The body of literature on injuries in ultimate is very limited compared to other popular sports. Despite conducting a comprehensive search of five major databases using a sensitive and inclusive search strategy (e.g., no restrictions on language or year of publication), relatively few records were identified. Although we identified a few additional potentially relevant articles through hand-searching, none of these were eligible for inclusion in this review. Our synthesis of the available epidemiological data was limited by the paucity, quality, and methodological heterogeneity of included studies, which, in turn, precluded a quantitative synthesis. The generalizability of the findings of this review may be limited because most of the available data are from adult athletes in the United States. It is possible that the injury problem in ultimate is different in other subpopulations (e.g., youth athletes and athletes in other geographical locations).

### 4.5. Recommendations for Future Research and Practice

Future studies investigating injuries in ultimate are strongly encouraged to adopt and implement definitions and recommendations outlined in the IOC consensus statement on sports injury and illness surveillance [19]. This includes, but is not necessarily limited to, clearly stating the operational injury and exposure definitions being used; adhering to classification systems (e.g., mechanism of injury, mode of onset, injury diagnoses); and determine injury severity by measuring actual time absent from participation in training or competition. To better facilitate comparisons by sex, future studies are recommended to report injury and exposure data by sex, whenever possible. Future studies are also strongly encouraged to investigate a wider array of potentially modifiable risk factors for injury in ultimate that may be targeted by subsequent injury prevention initiatives.

Ultimate coaches and players should be educated about injury risk management to enable them to prevent injuries from occurring in the first instance and to allow injured athletes to adequately recover from injury before returning to training and competition. It is recommended that an ongoing, long-term injury surveillance system is established and implemented to collect good-quality data that can be used for measuring the impact of any future injury prevention initiatives in ultimate. Ultimately, successful development and implementation of effective, sport-specific injury prevention strategies requires commitment and collaboration among stakeholders (e.g., coaches, athletes, athletic trainers, physiotherapists, and researchers). 

## 5. Conclusions

Despite being a non-contact sport with an emphasis on fair play, there is a substantial risk of injury in ultimate. The injury pattern in ultimate is similar to that in other team sports involving high-intensity running and active use of the upper limbs, with muscle strains and joint sprains to the knee, thigh, ankle, and shoulder areas being predominant. There is also a relatively high lifetime prevalence of concussion in ultimate. Development and implementation of effective, sport-specific injury prevention initiatives, including improved injury risk management and sport safety culture, should be a priority to reduce the burden of injury in ultimate. 

## Figures and Tables

**Table 1:** Characteristics of included studies.

Study	Country	Setting	Study Design	Study Period	Sample Size	Participants	Injury Definition	Number of Injuries	Outcome Measure	Quality
Marfleet 1991 [3]	United Kingdom, Germany, Belgium, Denmark, Norway	World championships (Competition)	Prospective cohort	1986 to 1990 (5 years)	N = 1000	Sex: Men’s and women’s (proportions NR) Age: Master’s, open’s, and junior (proportions NR) Experience: NR	NR	n = 485	NR	Moderate
Reynolds and Halsmer 2006 [29]	United States	Regional tournament (Competition)	Cross-sectional	2002	N = 135	Sex: 41% females, 59% males Age: 18–46 years Experience: 7.5 years	NR	NR	Lifetime prevalence: 100%	Good
Yen et al. 2010 [31]	United States	College championship (Competition)	Prospective cohort	2007 (3 days)	N = 705	Sex: 50% women’s teams, 50% men’s teams Age: NR Experience: NR	Injury time-out: any injury that required a player to miss part of the game or practice.	n = 107	Injury incidence rate: 68.0 per 1000 AEs (females), 110.0 per 1000 AEs (males)	Good
McElveen et al. 2014 [28]	United States	College league (Competition)	Prospective cohort	2011 to 2013 (2 years)	N = 553	Sex: NR Age: NR Experience: NR	Medical attention injury: any injury that occurred during participation in an intramural game and resulted in care by the intramural staff or more advanced care.	n = 6	Injury incidence proportion: 1.0 per 100 athletes (competition)	Poor
Akinbola et al. 2015 [25]	United States	University club sports (Competition and training)	Prospective cohort	2000 to 2012 (12 years)	N = 97	Sex: NR Age: NR Experience: NR	Medical attention injury: any injury for which an athlete sought medical attention at the Sports Clinic.	n = 143	12-year period prevalence: 100%	Moderate
Swedler et al. 2015 [30]	United States	College series (Competition and training)	Prospective cohort	2012 (4 months)	NR	Sex: 50% women’s teams, 50% men’s teams Age: NR Experience: NR	Injury time-out: any injury that required a player to miss part of the game or practice.	n = 1317	Injury incidence rate: 14.5 per 1000 AEs (competition), 10.1 per 1000 AEs (training)	Good
Kolodziej et al. 2017 [26]	Poland	Local club sports (Competition and training)	Cross-sectional	2016	N = 110	Sex: 33% females, 67% males Age: NR Experience: <1 year	NR	n = 408	Lifetime prevalence: 100%	Good
Lazar et al. 2018 [27]	United States	National teams and leagues (Competition and training)	Cross-sectional	2012	N = 787	Sex: 30% females, 70% males Age: NR Experience: 8.8 years	Concussion	n = 338	Lifetime prevalence: 26%	Good
Arthur-Banning et al. 2018 [34]	United States	University club sports (Competition and training)	Retrospective cohort	NR (2 years)	NR	Sex: NR Age: NR Experience: NR	Time-loss injury: incident that required medical attention and resulted in restriction to participation for at least one day.	NR	Injury incidence rate: 1.3 per 1000 AEs (competition, males), 0.4 per 1000 AEs (training, males), 9.2 per 1000 AEs (competition, females)	Moderate
Hess et al. 2020 [32]	United States, Canada	National league (Competition and training)	Prospective cohort	2017	NR	Sex: 100% men’s teams Age: NR Experience: NR	Injury time-out: any physical harm that happened while the player was participating in competition or practice and caused the player to miss part of a competition or practice.	n = 299	Injury incidence rate: 45.1 per 1000 AEs (competition), 20.0 per 1000 AEs (training)	Good
Brezinski et al. 2020 [33]	United States	University club sports (Competition)	Prospective cohort	2018 (16 weeks)	N = 69	Sex: 30% females, 70% males Age: NR Experience: NR	Time-loss injury: incident that required medical attention and resulted in restriction to participation for at least one day.	n = 18	Injury incidence rate: 11.1 per 1000 AEs (females), 9.6 per 1000 AEs (males)	Good

**Abbreviations:** NR, not reported; AE, athlete-exposure; CI, confidence interval.

**Table 2:** Frequency and proportion of injuries by body region and anatomical area ^a.^

Body Region/ Anatomical Area	Marfleet 1991 [3]		Yen et al. 2010 [31]		Akinbola et al. 2015 [25]		Swedler et al. 2015 [30] ^b^		Kolodziej et al. 2017 [26]		Hess et al. 2020 [32] ^c^	
	n	%	n	%	n	%	n	%	n	%	n	%
Head and Neck	32	6.7	15	15.0	-	-	139	10.9	63	15.4	10	3.5
Head	7	1.5	-	-	-	-	89	7.0	14	3.4	-	-
Neck	10	2.1	-	-	-	-	7	0.5	-	-	-	-
Other	15	3.1	-	-	-	-	43	3.4	49	12.0	-	-
Upper Limb	88	18.3	11	11.0	6	4.2	186	14.6	17	4.2	45	16.0
Shoulder	22	4.6	-	-	3	2.1	83	6.5	3	0.7	17	6.0
Upper arm	1	0.2	-	-	-	-	-	-	-	-	-	-
Elbow	23	4.8	-	-	-	-	17	1.3	5	1.2	11	3.9
Forearm	6	1.3	-	-	-	-	-	-	-	-	-	-
Wrist and hand	36	7.5	-	-	3	2.1	86	6.8	9	2.2	17	6.0
Trunk	37	7.7	12	12.0	11	7.7	68	5.3	108	53.4	16	5.7
Chest	9	1.9	-	-	-	-	-	-	-	-	-	-
Thoracic spine	28	5.8	-	-	-	-	68	5.3	64	15.7	-	-
Lumbar spine	-	-	-	-	11	7.7	-	-	44	10.8	-	-
Abdomen	4	0.8	-	-	-	-	-	-	110	27.0	-	-
Lower Limb	323	67.3	62	62.0	126	88.1	881	69.2	110	27.0	210	74.5
Hip and groin	10	2.1	-	-	10	7.0	85	6.7	6	1.5	13	4.6
Thigh	103	21.5	-	-	15	10.5	152	11.9	-	-	50	17.7
Knee	73	15.2	-	-	50	35.0	209	16.4	37	9.1	41	14.5
Lower leg	48	10.0	-	-	21	14.7	93	7.3	49	12.0	35	12.4
Ankle	59	12.3	-	-	30	21.0	265	20.8	17	4.2	58	20.6
Foot	30	6.3	-	-	-	-	77	6.0	1	0.2	13	4.6

^a.^ Proportions calculated from reported data with missing and unspecified injuries omitted. ^b.^ Missing (n = 6) and unspecified (n = 37) injuries omitted. ^c.^ Missing (n = 18) injuries omitted.

**Table 3:** Frequency and proportion of injuries by tissue type and pathology type ^a.^

Type of Tissue/Pathology	Marfleet. 1991 [3] ^b^		Swedler et al. 2015 [30] ^c^		Kolodziej et al. 2017 [26] ^d^		Hess et al. 2020 [32] ^e^	
	n	%	n	%	n	%	n	%
Muscle/Tendon	202	45.8	342	39.2	30	29.7	97	42.5
Muscle injury	179	40.6	319	36.5	30	29.7	86	37.7
Tendon rupture	23	5.2	23	2.6	-	-	11	4.8
Nervous	7	1.6	47	5.4	-	-	2	0.9
Brain/Spinal cord injury	6	1.4	47	5.4	-	-	2	0.9
Peripheral nerve injury	1	0.2	-	-	-	-	-	-
Bone	5	1.1	41	4.7	-	-	6	2.6
Fracture	5	1.1	41	4.7	-	-	6	2.6
Cartilage/Synovium/Bursa	23	5.2	7	0.8	-	-	4	1.8
Synovitis/Capsulitis	23	5.2	-	-	-	-	4	1.8
Ligament/Joint Capsule	38	8.6	246	28.2	71	70.3	62	27.2
Joint sprain	38	8.6	246	28.2	71	70.3	62	27.2
Superficial Tissues/Skin	166	37.6	190	21.8	-	-	57	25.0
Contusion (superficial)	110	24.9	140	16.0	-	-	46	20.2
Laceration	56	12.7	50	5.7	-	-	11	4.8

^a.^ Proportions calculated from reported data with missing and unspecified injuries omitted. ^b.^ Unspecified (n = 44) injuries omitted. ^c.^ Unspecified (n = 444) injuries omitted. ^d.^ Unspecified (n = 9) injuries omitted. ^e.^ Unspecified (n = 71) injuries omitted.

**Table 4:** Frequency and proportion of injuries by mechanism of injury ^a^.

Injury Mechanism	Yen et al. 2010 [31]		Swedler et al. 2015 [30] ^b^		Hess et al. 2020 [32] ^c^	
	n	%	n	%	n	%
Non-Contact	44	40.4	871	68.6	191	65.0
Direct Contact	65	59.6	399	31.4	103	35.0
With another athlete	43	39.4	387	30.5	101	34.4
With an object	22	20.2	12	0.9	2	0.7

^a.^ Proportions calculated from reported data with missing and unspecified injuries omitted. ^b.^ Missing (n = 32) and unspecified (n = 15) injuries omitted. ^c.^ Unspecified (n = 5) injuries omitted.

**Table 5:** Frequency and proportion of injuries by mode onset ^a,b^.

Mode of Onset	Swedler et al. 2015 [30] ^c^		Hess et al. 2020 [32]	
	n	%	n	%
Acute	1027	79.7	275	92.0
Sudden onset	1027	79.7	275	92.0
Repetitive	262	20.3	24	8.0
Sudden onset	-	-	-	-
Gradual onset	262	20.3	24	8.0

^a.^ Overuse/accumulation injuries were classified as repetitive and gradual onset; other injuries were classified as acute. ^b.^ Proportions calculated from reported data with missing injuries omitted. ^c.^ Missing (n = 28) injuries omitted.

## Supplementary Materials

The following are available online at https://www.mdpi.com/2075-4663/8/12/168/s1, Table S1: Full Search String for the MEDLINE Database; Table S2: Quality Assessment of Included Studies Using STROBE Statement Checklist; Figure S1: Graphical Overview for Evidence Reviews (GOfER) diagram; Video S1: Layouts executed from defensive and offensive positions.
