# Supplementary material for: Epidemiology of Injuries in Ultimate (Frisbee): A Systematic Review
Source: Sports (Basel). 2020 Dec 21;8(12):168. doi: 10.3390/sports8120168 (PMC7767421; doi:10.3390/sports8120168)
Supplement: Supplementary file 1 [file sports-08-00168-s001.zip › Supplementary Material - Figure S1.pdf]

Figure S1. Graphical Overview for Evidence Reviews (GOfER) diagram.

Study	Country	Setting	Design	Quality	Sample Size	Sex	Injuries	Body Region	Injury Type	Injury Mechanism
Marfleet 1991	Belgium<br>Denmark<br>Germany<br>Norway<br>UK	World championship (Competition)	Prospective cohort	Moderate	N=1000	NR	n=485	Head and neck 7%<br>Upper limb 18%<br>Trunk 8%<br>Lower limb 67%	Muscle/Tendon 46%<br>Ligament/Joint capsule 9%<br>Superficial tissues/Skin 38%<br>Other 8%	NR
Reynolds and Halsmer 2006	USA	Regional tournament (Competition)	Cross-sectional	Good	N=135	F M	NR	NR	NR	NR
Yen et al. 2010	USA	College championship (Competition)	Prospective cohort	Good	N=705	F M	n=107	Head and neck 15%<br>Upper limb 11%<br>Trunk 12%<br>Lower limb 62%	NR	Non-contact 40%<br>Indirect contact 0%<br>Direct contact 60%
McElveen et al. 2014	USA	College league (Competition)	Prospective cohort	Poor	N=553	NR	n=6	NR	NR	NR
Akinbola et al. 2015	USA	University sport clubs (Competition and training)	Prospective cohort	Moderate	N=97	NR	n=143	Head and neck 0%<br>Upper limb 4%<br>Trunk 8%<br>Lower limb 88%	NR	NR
Swedler et al. 2015	USA	College series (Competition and training)	Prospective cohort	Good	NR	F M	n=1317	Head and neck 11%<br>Upper limb 15%<br>Trunk 5%<br>Lower limb 69%	Muscle/Tendon 39%<br>Ligament/Joint capsule 28%<br>Superficial tissues/Skin 22%<br>Other 11%	Non-contact 69%<br>Indirect contact 0%<br>Direct contact 31%
Kolodziej et al. 2017	Poland	Local club sports (Competition and training)	Cross-sectional	Good	N=110	F M	n=408	Head and neck 15%<br>Upper limb 4%<br>Trunk 54%<br>Lower limb 27%	Muscle/Tendon 30%<br>Ligament/Joint capsule 70%<br>Superficial tissues/Skin 0%<br>Other 0%	NR
Lazar et al. 2018	USA	National teams and leagues (Competition and training)	Cross-sectional	Good	N=787	F M	n=338	Head and neck 100%	Concussion 100%	Non-contact 3%<br>Indirect contact 97%<br>Direct contact 0%
Arthur-Banning et al. 2018	USA	University club sports (Competition and training)	Retrospective cohort	Moderate	NR	NR	NR	NR	NR	NR
Hess et al. 2020	USA<br>Canada	National league (Competition and training)	Prospective cohort	Good	NR	F M	n=299	Head and neck 4%<br>Upper limb 16%<br>Trunk 6%<br>Lower limb 74%	Muscle/Tendon 43%<br>Ligament/Joint capsule 27%<br>Superficial tissues/Skin 25%<br>Other 5%	Non-contact 65%<br>Indirect contact 0%<br>Direct contact 35%
Brezinki et al. 2020	USA	University club sports (Competition and training)	Prospective cohort	Good	N=69	F M	n=18	NR	NR	NR

Abbreviations: USA, United States of America; UK, United Kingdom; F, Female; M, Male; NR, Not reported
