# Supplementary material for: Epidemiology of Injuries in Ultimate (Frisbee): A Systematic Review
Source: Sports (Basel). 2020 Dec 21;8(12):168. doi: 10.3390/sports8120168 (PMC7767421; doi:10.3390/sports8120168)
Supplement: Supplementary file 1 [file sports-08-00168-s001.zip › Supplementary Material - Table S1.pdf]

Table S1. Full Search String for the MEDLINE Database.

#	**Search String**
**1**	exp "Wounds and Injuries"/
**2**	injur*.mp.
**3**	wound*.mp.
**4**	trauma*.mp.
**5**	1 or 2 or 3 or 4
**6**	frisbee.mp.
**7**	(flying and (disc or disk)).mp. [mp=title, abstract, original title, name of substance word, subject heading word, floating sub-heading word, keyword heading word, protocol supplementary concept word, rare disease supplementary concept word, unique identifier, synonyms]
**8**	6 or 7
**9**	5 and 8
