# Supplementary material for: Epidemiology of Injuries in Ultimate (Frisbee): A Systematic Review
Source: Sports (Basel). 2020 Dec 21;8(12):168. doi: 10.3390/sports8120168 (PMC7767421; doi:10.3390/sports8120168)
Supplement: Supplementary file 1 [file sports-08-00168-s001.zip › Supplementary Material - Table S2.pdf]

Table S2. Quality Assessment of Included Studies Using STROBE Statement Checklist.

Item	Marfleet 1991	Reynolds and Halsmer 2006	Yen et al. 2010	McElveen et al. 2014	Akinbola et al. 2015	Swedler et al. 2015	Kolodziej et al. 2017	Lazar et al. 2018	Arthur-Banning et al. 2018	Hess et al. 2020	Brezinski et al. 2020
**Title and abstract**	✓	✓	✓	✓	✓	✓	✓	✓	✓	✓	✓
**Introduction**											
Background/rationale	✓	✓	✓	✓	✓	✓	✓	✓	✓	✓	✓
Objectives	✓	✓	✓	✓	✓	✓	✓	✓	✓	✓	✓
**Methods**											
Study design	–	✓	✓	✓	✓	✓	✓	✓	✓	✓	✓
Setting	✓	✓	✓	✓	✓	✓	✓	✓	✓	✓	✓
Participants	✓	✓	✓	✓	✓	✓	✓	✓	✓	✓	✓
Variables	–	✓	✓	–	✓	✓	✓	✓	✓	✓	✓
Data sources/measurement	✓	✓	✓	–	✓	✓	–	✓	✓	✓	✓
Bias	–	–	–	–	–	✓	–	–	–	✓	–
Study size	✓	✓	✓	–	–	✓	✓	✓	–	✓	✓
Quantitative variables	✓	–	–	–	–	✓	✓	–	✓	✓	✓
Statistical methods	–	✓	✓	✓	✓	✓	✓	✓	–	✓	✓
**Results**											
Participants	✓	✓	✓	–	✓	✓	✓	✓	–	–	–
Descriptive data	✓	–	✓	–	✓	✓	✓	✓	–	✓	✓
Outcome data	✓	✓	✓	✓	✓	✓	✓	✓	✓	✓	✓
Main results	✓	✓	✓	✓	✓	✓	✓	✓	✓	✓	✓
Other analysis	–	–	–	–	–	–	–	✓	–	✓	–
**Discussion**											
Key results	✓	✓	✓	✓	✓	✓	✓	✓	✓	✓	✓
Limitations	–	✓	✓	–	✓	✓	–	✓	✓	✓	✓
Interpretation	✓	✓	✓	✓	✓	✓	✓	✓	✓	✓	✓
Generalisability	–	–	–	–	✓	–	–	–	–	✓	✓
**Other information**											
Funding	–	–	✓	–	–	–	–	–	–	–	–
**Percentage fulfilled items**	64	73	82	50	77	86	73	82	64	91	82
